# High expression of DNA damage-inducible transcript 4 (DDIT4) is associated with advanced pathological features in the patients with colorectal cancer

**DOI:** 10.1038/s41598-021-92720-z

**Published:** 2021-07-01

**Authors:** Fahimeh Fattahi, Leili Saeednejad Zanjani, Zohreh Habibi Shams, Jafar Kiani, Mitra Mehrazma, Mohammad Najafi, Zahra Madjd

**Affiliations:** 1 Oncopathology Research Center, Iran University of Medical Sciences (IUMS), Tehran, Iran; 2 Department of Molecular Medicine, Faculty of Advanced Technologies in Medicine, Iran University of Medical Sciences, Tehran, Iran; 3 Department of Pathology, Iran University of Medical Sciences, Tehran, Iran; 4 Biochemistry Department, Faculty of Medical Sciences, Iran University of Medical Sciences, Tehran, Iran

**Keywords:** Tumour biomarkers, Colorectal cancer, Bioinformatics, Immunological techniques, Medical research

## Abstract

DNA damage-inducible transcript 4 (DDIT4) is induced in various cellular stress conditions. This study was conducted to investigate expression and prognostic significance of DDIT4 protein as a biomarker in the patients with colorectal cancer (CRC). PPI network and KEGG pathway analysis were applied to identify hub genes among obtained differentially expressed genes in CRC tissues from three GEO Series. In clinical, expression of DDIT4 as one of hub genes in three subcellular locations was evaluated in 198 CRC tissues using immunohistochemistry method on tissue microarrays. The association between DDIT4 expression and clinicopathological features as well as survival outcomes were analyzed. Results of bioinformatics analysis indicated 14 hub genes enriched in significant pathways according to KEGG pathways analysis among which DDIT4 was selected to evaluate CRC tissues. Overexpression of nuclear DDIT4 protein was found in CRC tissues compared to adjacent normal tissues (*P* = 0.003). Furthermore, higher nuclear expression of DDIT4 was found to be significantly associated with the reduced tumor differentiation and advanced TNM stages (all, *P* = 0.009). No significant association was observed between survival outcomes and nuclear expression of DDIT4 in CRC cases. Our findings indicated higher nuclear expression of DDIT4 was significantly associated with more aggressive tumor behavior and more advanced stage of disease in the patients with CRC.

## Introduction

Colorectal cancer (CRC) as a heterogeneous disease is considered as the second leading cause of cancer-related deaths worldwide^1^. Molecular heterogeneity of CRC is due to genetic modifications commonly driving advancement in cancer and development of the disease^2,3^. Overall survival varies significantly in the patients with CRC depending on stage of the disease at the time of diagnosis and treatment decisions^4–6^. Despite the recent advances in screening and treatment of CRC, there is still a need to identify new markers for early diagnosis and prognosis in order to improve treatment decisions^6^. Therefore, diagnostic and prognostic biomarkers are required that could be more clinically applicable rather than conventional biomarkers^7^ and stratify the patients with CRC for cancer therapy^6,8,9^. In this regard, studying character and frequency of genetic and epigenetic alterations in cancer has been used as a great tool to investigate and identify those biomarkers which are linked to tumor development and medicine arrangements^3,9^.

Omics technologies have been used in order to characterize molecular features of tumor cells and their functional irregularities in cancer findings of which have been utilized in clinical settings to help cancer treatment^10^. Bioinformatics and computational biology are also necessary for analysis of Omics data and discovering biomarkers that will become an important part of medical research as well as clinical routine^11^. Therefore, in the present study, bioinformatics tools were applied to identify hub genes among the differentially expressed genes (DEGs) influencing CRC from transcriptome data, which led to identification of DNA damage-inducible transcript 4 (DDIT4) to investigate in CRC tissues as prognostic biomarker.

DDIT4 also known as a regulated in development and DNA damage response 1 (REDD1) protein and hypoxia-inducible factor 1 (HIF1)-responsive protein RTP801 (RTP801) was discovered and cloned in 2002^12,13^. Generally, DDIT4 is rapidly induced in various cellular stresses^12,14^, such as hypoxia^15,16^, heat shock^17^, endoplasmic reticulum stress, and chemical molecules^18^. Most reports on DDIT4 function have indicated that DDIT4 protein suppresses mammalian target of rapamycin complex 1 (mTORC1) and regulates cell growth, tumorigenesis, cell aging, and autophagy^12,14,19–21^.

Upregulation of DDIT4 has been shown to promote cell proliferation, reduce apoptotic rate, and S phase arrest in gastric epithelial cells^22^. Moreover, upregulation of DDIT4 protein has been found to be associated with the decreased expression of pro-apoptotic proteins and at the same time the increased levels of anti-apoptotic proteins after inducing activity of RAS oncogene in ovarian epithelial cells^23,24^.

Prominently, the previous studies have revealed that dysregulation of DDIT4 occurs in various human cancers with paradoxical roles. A number of studies have attributed DDIT4 to tumor suppressor process, through suppression of mTORC1 in CRC^25^, breast cancer^26^, sporadic clear cell renal cell carcinoma (ccRCC)^27^, and non-small cell lung cancer^28^. While as an oncogene, upregulation of DDIT4 contributes to reduction of apoptosis processes, promotion of proliferation, migration, and invasion of cancer cells in in-vitro and in-vivo cancer studies^22,29–31^. High expression levels of DDIT4 protein have been observed in ovarian cancer (OC)^31^, bladder urothelial carcinoma (BUC)^30^, ccRCC (patients with von Hippel Lindau-deficient)^27^, and gastric cancer (GC)^22^ tissues compared to adjacent normal tissues. Increased expression of DDIT4 has been remarked as a prognosis factor in the patients with OC and BUC^30–32^. Additionally, DDIT4 gene is introduced as a cell intrinsic regulator for cancer therapy resistance in some cancers like brain, lung and gastric because it confers protection of tumor cells from therapy^19,22,33^. In contrast, in CRC, therapy with baicalein and polyisoprenylated benzophenones as anticancer agents in in-vitro indicated upregulation of DDIT4 associated with growth inhibition^25,34^.

In the present study, publicly available transcriptomic series (GSE74602, GSE110223, and GSE110224) were applied to detect genes that were differentially expressed in tumor tissues compared to adjacent normal tissues from the CRC patients. For identifying hub genes based on protein information, a protein–protein interaction (PPI) network was created for common upregulated DEGs among these three series. Then, Kyoto encyclopedia of genes and genomes (KEGG) pathway enrichment analysis was done in order to better understand the pathways in which hub genes are involved. The bioinformatics analysis and literature review of these hub genes led to selection of DDIT4 as a biomarker in CRC tissues. Besides, in our other study, we have evaluated and detected mRNA expression levels of DDIT4 as a predictor biomarker for advanced disease in fresh CRC tissue samples^35^, herein, expression of DDIT4 protein was further confirmed as a biomarker in clinical CRC samples. Expression levels and localization of DDIT4 protein, for the first time were investigated in nucleus, cytoplasm, and plasma membrane for a series of formalin-fixed paraffin-embedded (FFPE) tissues from CRC patients using the immunohistochemistry (IHC) method on tissue microarrays (TMAs). Then, the associations between expression levels of DDIT4 protein at different subcellular locations with clinicopathological features as well as survival outcomes were analyzed.

## Results

### Bioinformatics approach

#### Identification of the upregulated DEGs and PPI network

**Figure 1:**
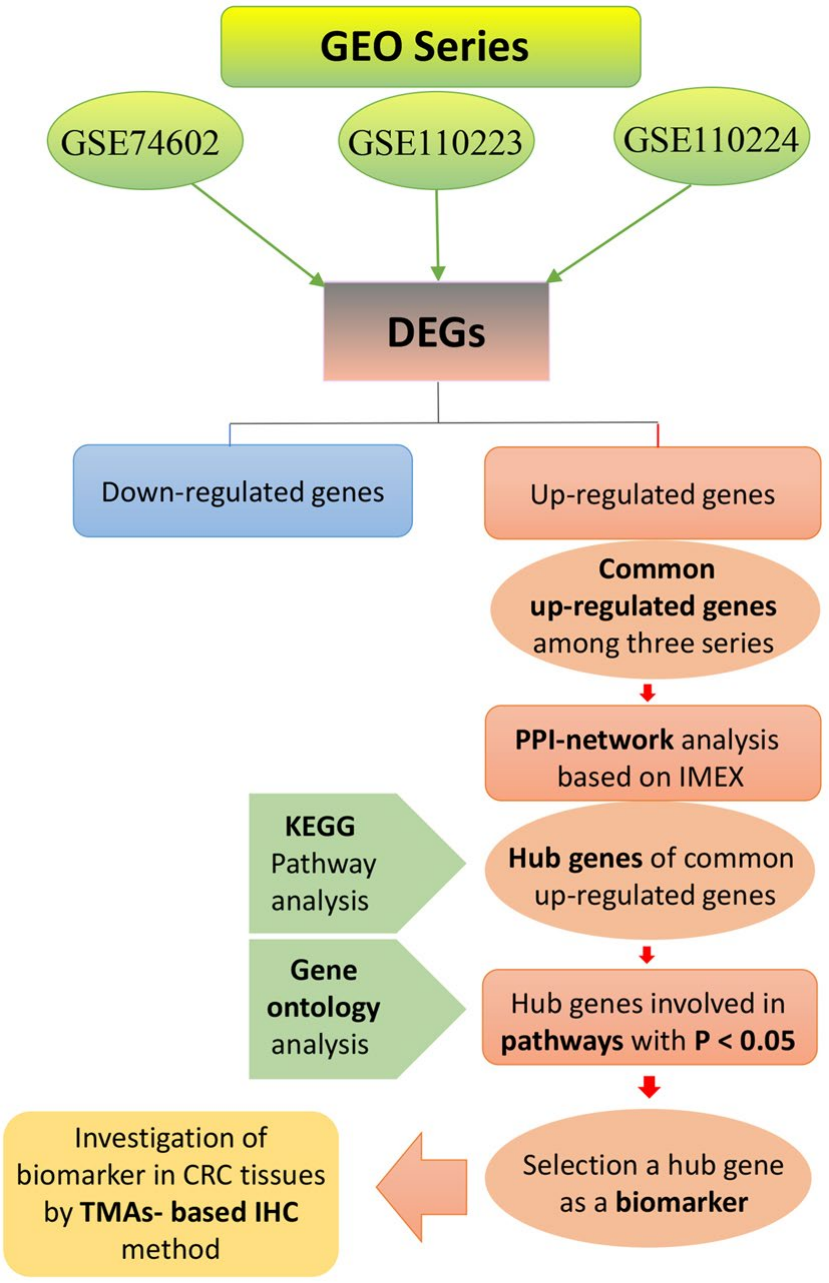
The workflow of the present study. This flowchart is a schematic overview of the bioinformatics analysis for selection of biomarker to investigate in CRC samples using the tissue microarrays-based immunohistochemistry (TMAs-based IHC).

**Figure 2:**
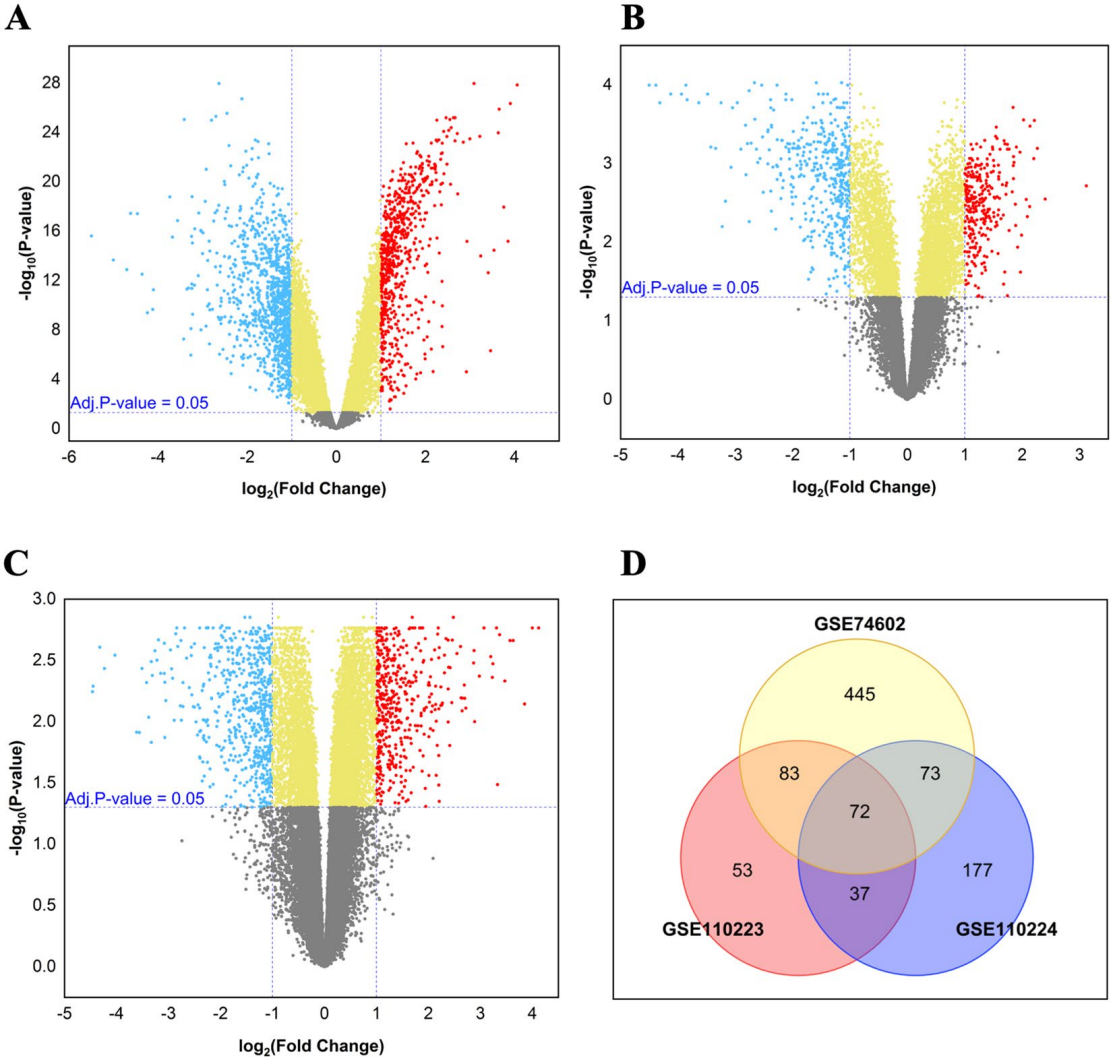
Identification of the differentially expressed genes (DEGs) in tumor tissues compared to adjacent normal tissues. Volcano plots of the gene expression profile from the (**A**) GSE74602, (**B**) GSE110223, and (**C**) GSE110224 series. The red dots at the top right represent the upregulated DEGs and the blue dots at the top left represent the downregulated DEGs (log FC ≥  ± 1, adjusted *P* value < 0.05). (**D**) Venn diagram displayed common upregulated DEGs among three series for subsequent analysis.

The workflow of our research is presented in Fig. 1. Based on the preset criteria of log FC ≥  ± 1.0 and adjusted *P* value < 0.05, 1672 DEGs (673 upregulated and 999 downregulated), 589 DEGs (245 upregulated and 344 downregulated), and 814 DEGs (359 upregulated and 455 downregulated) were extracted from GSE74602, GSE110223, and GSE110224 series, respectively, using volcano plots as shown in Fig. 2A–C. Further investigation of the obtained upregulated DEGs from three series using Venn diagram analysis displayed that there were 72 common upregulated DEGs between these series (Fig. 2D and Supplementary Table 1). Despite the most of DEGs in the current study were in line with our previous results in other CRC series of GEO^36^, we rechecked 72 common upregulated DEGs from this analysis with obtained common DEGs of merged three series (GSE74602, GSE110223, and GSE110224) by MINT tool method (Supplementary Fig. S1 and Table S1). Comparison between these DEGs by MINT and 72 common upregulated genes showed that total 72 common upregulated genes exist in common upregulated DEGs by MINT method.

**Figure 3:**
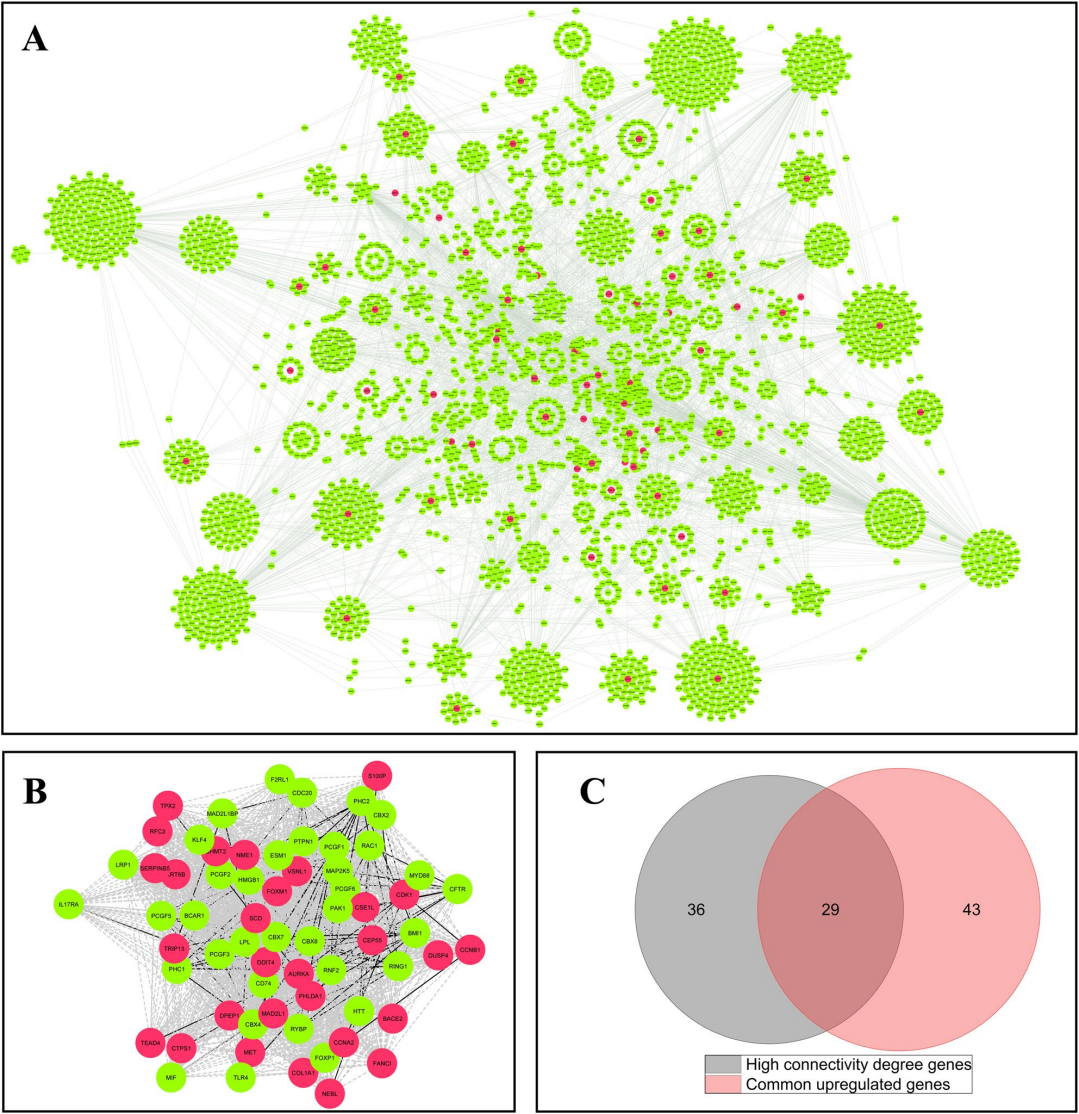
Protein–protein interaction (PPI) analysis based on IMEX database for common upregulated DEGs and detection of hub genes. (**A**) The nodes represent genes and edges represent the interactions between genes. The red nodes signify 72 common upregulated DEGs that are in the interaction with each other and other genes (green nodes). (**B**) PPI network analysis revealed the genes with high connectivity degree (> 75 percentile for the genes with degree > 10) by cyto-Hubba plug-in using Cytoscape software. The dashed lines indicate the connection of genes that are not direct. (**C**) The genes with high connectivity degree that overlapped with common upregulated DEGs were extracted as hub genes by Venn diagram.

For investigating all the protein interactions available for 72 common upregulated DEGs, a PPI network was constructed for these genes according to IMEX database, which included 3831 nodes and 9911 edges (Fig. 3A). Sixty-five genes with high connectivity degree were extracted from this network by cyto-Hubba in which 29 genes overlapped with 72 common upregulated DEGs (Fig. 3B,C). Therefore, these 29 genes were considered as hub genes of common upregulated DEGs for subsequent analysis (Supplementary Table 1).

#### KEGG pathway and gene ontology (GO) analysis for hub genes

**Figure 4:**
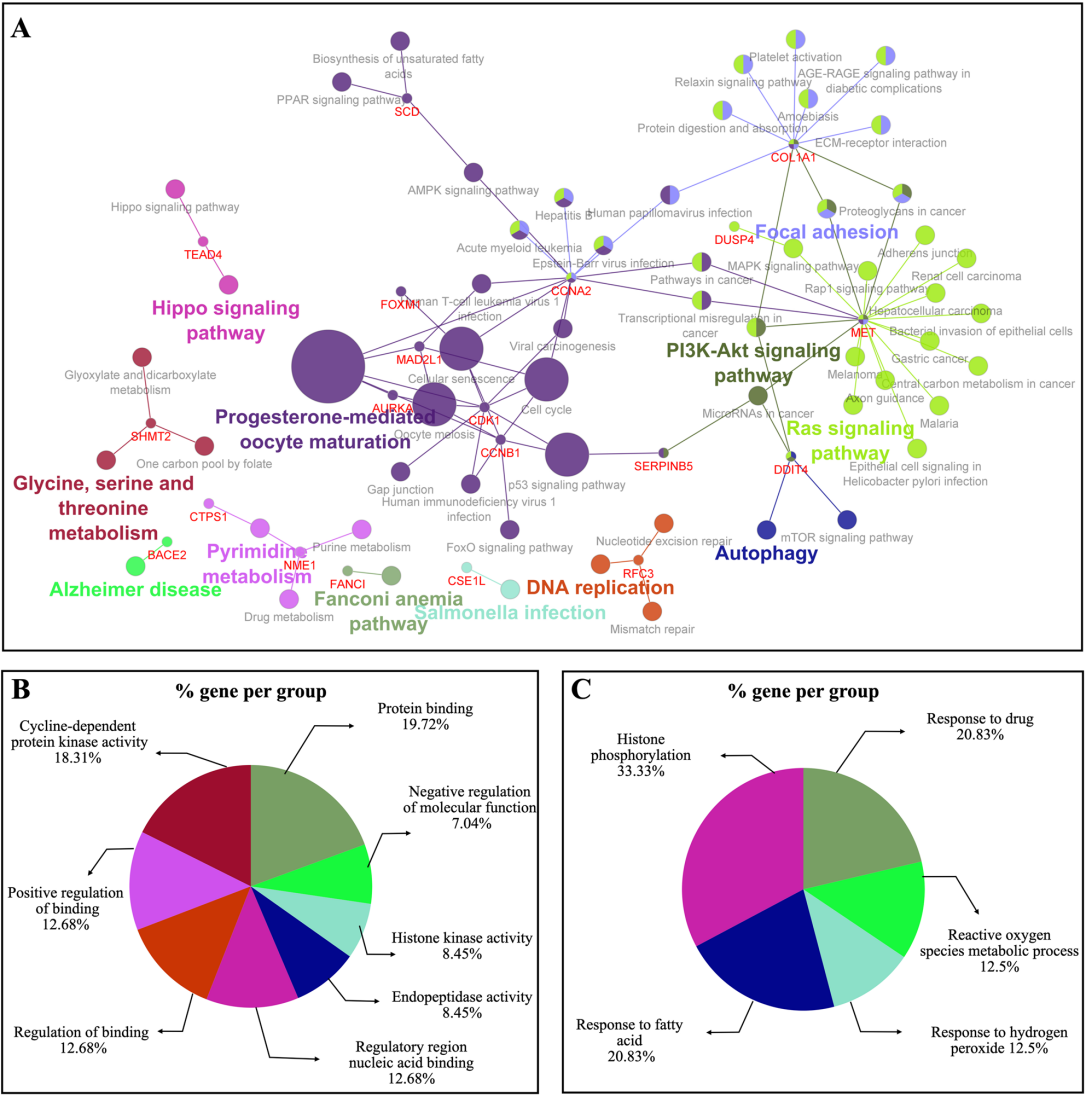
KEGG pathways and gene ontology (GO) analysis for hub genes in Cytoscape software by ClueGO plug-in. (**A**) KEGG pathway analysis for 29 obtained hub genes of protein–protein interaction network. (**B**) Gene ontology analysis for domains of molecular function (MF) and (**C**) biological process (BP) for 14 hub genes involved in KEGG pathways with *P* value < 0.05.

The KEGG pathway analysis for 29 hub genes indicated that these genes were involved and clustered in different pathways, such as ‘progesterone-mediated oocyte maturation’, ‘cell cycle’ and ‘PI3K-Akt signaling pathway’ as summarized in Fig. 4A. (Supplementary Table 2). Moreover, 14 hub genes were found in 10 pathway terms with *P* < 0.05 (Table 1) that GO enrichment analysis was performed in order to obtain more comprehensive and deep understanding of the biological process and function of these hub genes. GO enrichment analysis in MF domain indicated that most 14 hub genes were clustered in functional groups of ‘protein binding’ and ‘cyclin-dependent protein kinase activity’ (Fig. 4B). These hub genes were mainly enriched in ‘histone phosphorylation’ and ‘response to drug’ groups of BP domain (Fig. 4C). Also, KEGG pathways involved in CRC diseases were obtained from the KEGG DISEASE Database, which included ‘mucin type O-glycan biosynthesis’, ‘colorectal cancer pathway’, and ‘microRNAs in cancer’ pathways as summarized in Supplementary Fig. S2. Then, accurate scrutiny of hub genes҆ pathways displayed those 3 hub genes (MET, DDIT4, and SERPINB5) were involved in ‘microRNAs in cancer’ pathway, a part of pathways in CRC diseases based on KEGG database. Finally, literature review and enrichment analysis led to selection of DDIT4 to evaluate protein expression level of this marker in CRC tissues. According to the KEGG pathway analysis, DDIT4 was found to be related to PI3k-Akt/mTOR signaling and microRNAs in cancer pathway, as a part of CRC disease using the KEGG DISEASE Database. PI3k-Akt/mTOR signaling is frequently deregulated in human cancer that is one of the primary mechanisms for sustaining tumor outgrowth and metastasis. Targeting these pathways have been considered as a strategy for cancer therapy^37,38^. DDIT4 was participated in important BP and MF, such as cell death, response to drug, protein binding, and cellular response to stress in the cell. Moreover, our review of literature indicated that DDIT4 protein expression was not evaluated in CRC patients with various stages by IHC.

**Table 1:** Hub genes involved in pathways with *P* value < 0.05 based on KEGG pathway analysis.

KEGG number	KEGG pathways	Gene names	*P* value
KEGG:04914	Progesterone-mediated oocyte maturation	AURKA, CCNA2, CCNB1, CDK1, MAD2L1	< 0.00001
KEGG:04110	Cell cycle	CCNA2, CCNB1, CDK1, MAD2L1	< 0.001
KEGG:04114	Oocyte meiosis	AURKA, CCNB1, CDK1, MAD2L1	< 0.001
KEGG:04218	Cellular senescence	CCNA2, CCNB1, CDK1, FOXM1	< 0.001
KEGG:04115	P53 signaling pathway	CCNB1, CDK1, SERPINB5	< 0.001
KEGG:00240	Pyrimidine metabolism	CTPS1, NME1	< 0.01
KEGG:04152	AMPK signaling pathway	CCNA2, SCD	0.035
KEGG:05206	MicroRNAs in cancer	DDIT4, MET, SERPINB5	0.040
KEGG:04151	PI3K-Akt signaling pathway	COL1A1, DDIT4, MET	0.045
KEGG:00670	One carbon pool by folate	SHMT2	0.048

Subcellular localization study for DDIT4 showed that this marker is mostly enriched in the cytosol and cell nucleus and also to some extent; it is found in the plasma membrane and extracellular environment based on COMPARTMENTS database.

### Findings related to the study population

#### Baseline characteristics of the study population

A total of 198 samples from CRC population were included in the current study, in which 104 (52.5%) samples belonged to males and 94 (47.5%) of them were from females with a male/female ratio of 1.1. Mean age of the patients was equal to 59 (SD = 13) years old, (ranging from 25 to 88); 98 (49.5%) patients were younger than 59 and 100 (50.5%) of them were over 59 years old. Tumor size, ranging from 1 to 24 cm was categorized based on mean size into two groups: Group 1: ≤ 5 cm [134 cases (67.7%)] and Group 2: > 5 cm [64 cases (32.3%)]. In this study, 90 (45.5%) patients had well differentiated, 95 (48.0%) patients had moderately differentiated, and 13 (6.6%) of them had poorly differentiated. Moreover, 31 (15.7%) cases were at stage I of the disease, 81 (40.9%) cases were at stage II, 79 (39.9%) cases were at stage III, and 7 (3.5%) cases were at stage IV according to TNM stage system. Vascular and perineural invasion were observed in 33 (16.7%) and 48 (24.2%) of the patients with CRC, respectively while lymph node metastasis was found in 84 (42.4%) cases.

#### Expression and localization of DDIT4 in CRC tissues

**Figure 5:**
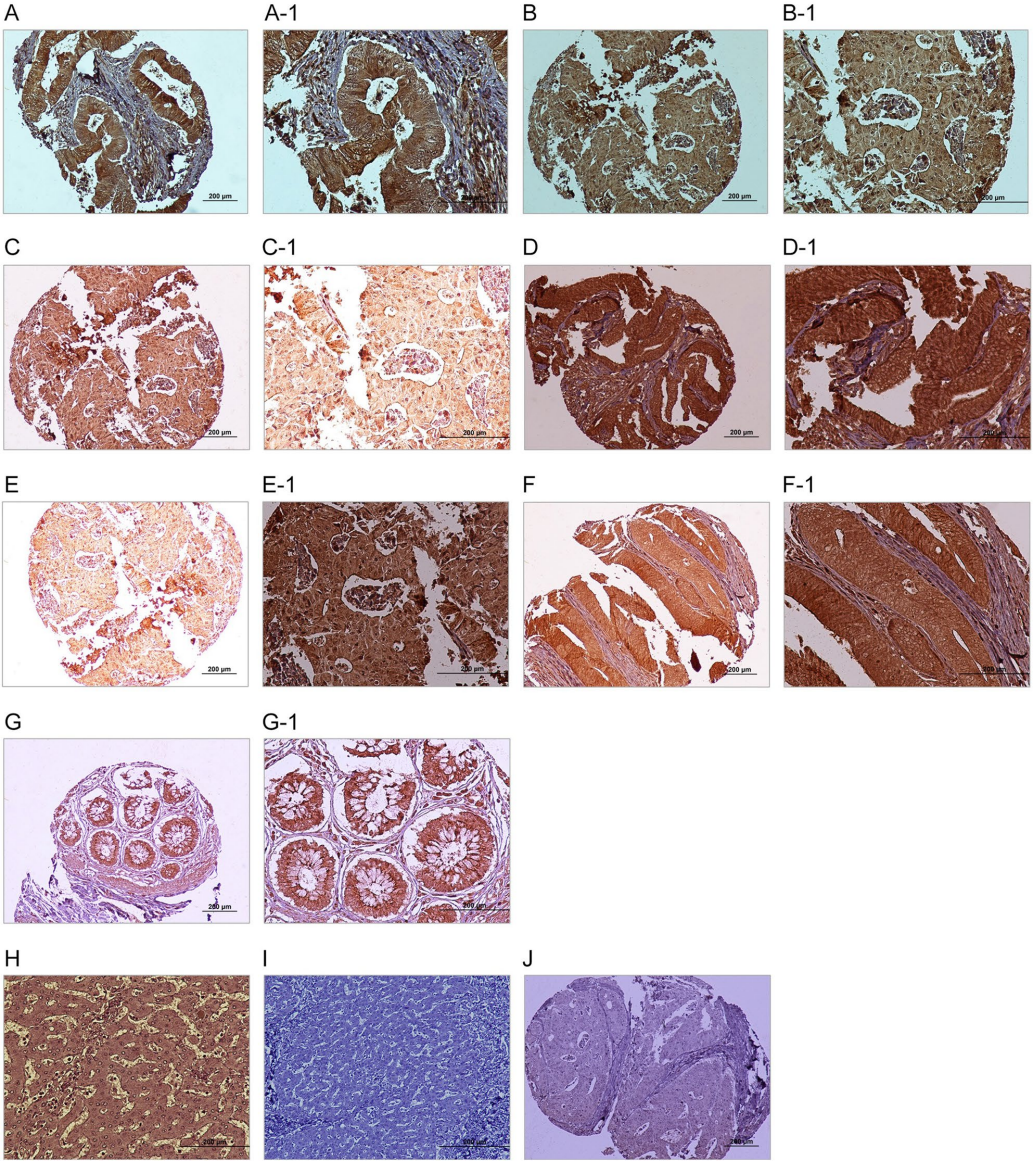
Immunohistochemical analysis of DDIT4 expression in colorectal cancer (CRC) samples. Nuclear expression of DDIT4 in CRC: (**A**, **A-1**) low expression and (**B**, **B-1**) high expression. Cytoplasmic expression of DDIT4 in CRC: (**C**, **C-1**) low expression and (**D**, **D-1**) high expression. Membranous expression of DDIT4 in CRC: (**E**, **E1**) low expression and (**F**, **F1**) high expression. (**G**, **G1**) DDIT4 expression in adjacent normal tissue. (**H**) DDIT4 expression in normal human liver as a positive control, (**I**) DDIT4 expression in normal human liver as a negative control, and (**J**) Isotype control. (Figures shown with magnification of 100 × and 200 ×).

For estimating expression levels of DDIT4, three scoring systems were employed incorporating intensity of staining, percentage of positive tumor cells, and the H-score. Furthermore, expression of DDIT4 was detected at different subcellular locations including nucleus, cytoplasm, and plasma membrane in both CRC tumor cells and adjacent normal tissues by IHC analysis. However, a significant overexpression of DDIT4 at nucleus was observed in tumor cells compared to adjacent normal tissue samples (*P* = 0.003). This comparison was not statistically significant in terms of cytoplasmic and membranous expression of DDIT4. Measurement of staining based on the median H-score as the cut-off was done for lower DDIT4 expression versus higher DDIT4 expression in the nucleus, cytoplasm, and plasma membrane of cells. Expression of DDIT4 protein in CRC and adjacent normal tissue samples are exhibited in Fig. 5A–G1. Also, DDIT4 expression was observed with various intensities amongst the CRC tissues in various locations (Table 2).

**Table 2:** Nuclear, cytoplasmic, and membranous DDIT4 expression in colorectal cancer (CRC) tissues and their adjacent normal tissue samples (Intensity of staining, percentage of positive tumor cells, and H-score).

Scoring system	Nuclear DDIT4 N (%)	Adjacent normal N (%)	*P* value	Cytoplasmic DDIT4 N (%)	Adjacent normal N (%)	*P* value	Membranous DDIT4 N (%)	Adjacent normal N (%)	*P* value
**Intensity of staining**			***0.004***			***0.023***			***0.013***
No staining (0)	0 (0.0)	1 (2.6)		0 (0.0)	0 (0.0)		2 (1.0)	0 (0.0)	
Weak (+ 1)	6 (3.0)	1 (2.6)		12 (6.1)	7 (17.9)		6 (3.0)	2 (5.1)	
Moderate (+ 2)	86 (43.4)	26 (66.7)		134 (67.7)	26 (66.7)		101 (51.0)	28 (71.8)	
Strong (+ 3)	106 (53.5)	11 (28.1)		52 (26.2)	6 (15.4)		89 (45.0)	9 (23.1)	
**Percentage of positive tumor cells**			0.781			0.533			0.086
< 25%	1 (0.5)	1 (2.6)		0 (0.0)	0 (0.0)		4 (2.0)	0 (0.0)	
25–50%	4 (2.0)	1 (2.6)		6 (3.0)	3 (7.7)		7 (3.5)	1 (2.6)	
51–75%	53 (26.8)	10 (25.6)		73 (36.9)	14 (35.9)		69 (34.8)	9 (23.1)	
> 75%	140 (70.7)	27 (69.2)		119 (60.1)	22 (56.4)		118 (59.6)	29 (74.4)	
**H-score cut off**			***0.003***			0.142			0.211
Low	93 (47.0)	22 (56.4)		120 (60.6)	25 (64.1)		115 (58.1)	31 (79.5)	
High	105 (53.0)	17 (43.6)		78 (39.4)	14 (35.9)		83 (41.9)	8 (20.5)	
Total	198	39		198	39		198	39	

*P value* is based on Mann–Whitney *U* test.

*H-score* histological score.

Besides, human normal liver tissue as a positive control exhibited moderate staining in cytoplasm of all the hepatocyte and bile duct cells as well as nucleus of a few cells lining the sinusoids and results related to positive, negative, and isotype controls are shown in Fig. 5H–J.

#### Association between expressions of DDIT4 and clinicopathological features

Nuclear and cytoplasmic expressions of DDIT4 were observed in all CRC samples while membranous expression of DDIT4 was not found in 2 (1%) cases of total patients with CRC. In this study, Pearson's chi-squared test was used to investigate the association between expression levels of DDIT4 and various clinicopathological features in the patients with CRC. The results of TMA-based IHC analysis were prepared according to the patients҆ clinicopathological features and are presented in Tables 3, 4, and 5 based on subcellular locations of DDIT4 marker staining. The results of Pearson’s chi-square test revealed that nuclear expression of DDIT4 in terms of intensity of staining (*P* = 0.031) and H-score (*P* = 0.009) had a significant association with tumor differentiation. In addition, a significant association was found between nuclear expression of DDIT4 and TNM stages (intensity of staining; (*P* = 0.013) and H-score [(*P* = 0.009), respectively]. The Spearman’s correlation test indicated a significant direct correlation between nuclear expression of DDIT4 with tumor differentiation and TNM stages (*P* < 0.05).

**Table 3:** The association between nuclear DDIT4 expression and clinicopathological features of colorectal cancer (CRC) samples (Intensity of staining and H-score).

Patients and tumor characteristics	Total samples N (%)	Intensity of staining N (%)				*P* value	H-score (cut off = 210) N (%)		*P* value
		0 (Negative)	1+ (Weak)	2+ (Moderate)	3+ (Strong)		Low (≤ 210)	High (> 210)	
**Mean age, years (Range)**	59 (25–88)					0.991			0.769
≤ Mean age	98 (49.5)	0 (0.0)	3 (50.0)	43 (50.0)	52 (49.1)		45 (48.4)	53 (50.5)	
> Mean age	100 (50.5)	0 (0.0)	3 (50.0)	43 (50.0)	54 (50.9)		48 (51.6)	52 (49.5)	
**Gender**						0.760			0.966
Male	104 (52.5)	0 (0.0)	4 (66.7)	44 (51.2)	56 (52.8)		49 (52.7)	55 (52.4)	
Female	94 (47.5)	0 (0.0)	2 (33.3)	42 (48.8)	50 (47.2)		44 (47.3)	50 (47.6)	
(Male/Female)	1.1	-	2	1.0	1.1		1.1	1.1	
**Mean tumor size (cm)**	5					0.159			0.985
≤ Mean	134 (67.7)	0 (0.0)	2 (33.3)	61 (70.9)	71 (67.0)		63 (67.7)	71 (67.6)	
> Mean	64 (32.3)	0 (0.0)	4 (66.7)	25 (29.1)	35 (33.0)		30 (32.3)	34 (32.4)	
**Tumor differentiation**						***0.031***			***0.009***
Well	90 (45.5)	0 (0.0)	2 (33.3)	50 (58.1)	38 (35.8)		53 (57.0)	37 (35.2)	
Moderately	95 (48.0)	0 (0.0)	4 (66.7)	31 (36.0)	60 (56.6)		35 (37.6)	60 (57.1)	
Poorly	13 (6.6)	0 (0.0)	0 (0.0)	5 (5.8)	8 (7.5)		5 (5.4)	8 (7.6)	
**TNM stages**						***0.013***			***0.009***
I	31 (15.7)	0 (0.0)	0 (0.0)	10 (11.6)	21 (19.8)		11 (11.8)	20 (19.0)	
II	81 (40.9)	0 (0.0)	3 (50.0)	46 (53.5)	32 (30.2)		48 (51.6)	33 (31.4)	
III	79 (39.9)	0 (0.0)	3 (50.0)	25 (29.1)	51 (48.1)		29 (31.2)	50 (47.6)	
IV	7 (3.5)	0 (0.0)	0 (0.0)	5 (5.8)	2 (1.9)		5 (5.4)	2 (1.9)	
**Vascular invasion (VI)**						0.874			0.848
Present	33 (16.7)	0 (0.0)	1(16.7)	13 (15.1)	19 (17.9)		15 (16.1)	18 (17.1)	
Absent	165 (83.3)	0 (0.0)	5 (83.3)	73 (84.9)	87 (82.1)		78 (83.9)	87 (82.9)	
**Lymph node invasion (LNI)**						0*.*343			0.199
Involved	84 (42.4)	0 (0.0)	2 (33.3)	32 (37.2)	50 (47.2)		35 (37.6)	49 (46.7)	
None	114 (57.6)	0 (0.0)	4 (66.7)	54 (62.8)	56 (52.8)		58 (62.4)	56 (53.3)	
**Perineural invasion (PI)**						0.314			0.629
Involved	48 (24.2)	0 (0.0)	3 (50.0)	21 (24.4)	24 (22.6)		24 (25.8)	24 (22.9)	
None	150 (75.8)	0 (0.0)	3 (50.0)	65 (75.6)	82 (77.4)		69 (74.2)	81 (77.1)	
**Distant metastasis**						0.500			0.989
Present	28 (14.1)	0 (0.0)	0 (0.0)	11 (25.6)	17 (26.2)		12 (25.0)	16 (25.0)	
Absent	84 (42.4)	0 (0.0)	4 (100.0)	32 (74.4)	48 (73.8)		36 (75.0)	48 (75.0)	
**Tumor recurrence**						0.381			0.533
Yes	29 (14.6)	0 (0.0)	0 (0.0)	10 (23.3)	19 (29.2)		11 (22.9)	18 (28.1)	
No	83 (41.9)	0 (0.0)	4 (100.0)	33 (76.7)	46 (70.8)		37 (77.1)	46 (71.9)	

*P value*; Pearson’s χ^2^ test, and Values in bold are statistically significant.

*H-score* histological score.

**Table 4:** The association between cytoplasmic DDIT4 expression and clinicopathological features of colorectal cancer (CRC) samples (Intensity of staining and H-score).

Patients and tumor characteristics	Total samples N (%)	Intensity of staining N (%)				*P* value	H-score (cut off = 180) N(%)		*P* value
		0 (Negative)	1+ (Weak)	2+ (Moderate)	3+ (Strong)		Low (≤ 180)	High (> 180)	
**Mean age, years (Range)**	59 (25–88)					0.187			0.640
≤ Mean age	98 (49.5)	0 (0.0)	8 (66.7)	69 (51.5)	21 (40.4)		61 (50.8)	37 (47.4)	
> Mean age	100 (50.5)	0 (0.0)	4 (33.3)	65 (48.5)	31 (59.6)		59 (49.2)	41 (52.6)	
**Gender**						0.557			0.377
Male	104 (52.5)	0 (0.0)	8 (66.7)	68 (50.7)	28 (53.8)		60 (50.0)	44 (56.4)	
Female	94 (47.5)	0 (0.0)	4 (33.3)	66 (49.3)	24 (46.2)		60 (50.0)	34 (43.6)	
(Male/Female)	1.1	-	2.0	1.0	1.1		1.0	1.2	
**Mean tumor size (cm)**	5					0.679			0.578
≤ Mean	134 (67.7)	0 (0.0)	9 (75.0)	92 (68.7)	33 (63.5)		83 (69.2)	51 (65.4)	
> Mean	64 (32.3)	0 (0.0)	3 (25.0)	42 (31.3)	19 (36.5)		37 (30.8)	27 (34.6)	
**Tumor differentiation**						0.554			0.987
Well	90 (45.5)	0 (0.0)	8 (66.7)	60 (44.8)	22 (42.3)		54 (45.0)	36 (46.2)	
Moderately	95 (48.0)	0 (0.0)	4 (33.3)	64 (47.8)	27 (51.9)		58 (48.3)	37 (47.4)	
Poorly	13 (6.6)	0 (0.0)	0 (0.0)	10 (7.5)	3 (5.8)		8 (6.7)	5 (6.4)	
**TNM stages**						0.0466			0.375
I	31 (15.7)	0 (0.0)	2 (16.7)	21 (15.7)	8 (15.4)		15 (12.5)	16 (20.5)	
II	81 (40.9)	0 (0.0)	5 (41.7)	60 (44.8)	16 (30.8)		53 (44.2)	28 (35.9)	
III	79 (39.9)	0 (0.0)	5 (41.7)	47 (35.1)	27 (51.9)		47 (39.2)	32 (41.0)	
IV	7 (3.5)	0 (0.0)	0 (0.0)	6 (4.5)	1 (1.9)		5 (4.2)	2 (2.6)	
**Vascular invasion (VI)**						0.207			0.696
Present	33 (16.7)	0 (0.0)	3 (25.0)	18 (13.4)	12 (23.1)		21 (17.5)	12 (15.4)	
Absent	165 (83.3)	0 (0.0)	9 (75.0)	116 (86.6)	40 (76.9)		99 (82.5)	66 (84.6)	
**Lymph node invasion (LNI)**						0.106			0.979
Involved	84 (42.4)	0 (0.0)	6 (50.0)	50 (37.3)	28 (53.8)		51 (42.5)	33 (42.3)	
None	114 (57.6)	0 (0.0)	6 (50.0)	84 (62.7)	24 (46.2)		69 (57.5)	45 (57.7)	
**Perineural invasion (PI)**						0.818			0.517
Involved	48 (24.2)	0 (0.0)	2 (16.7)	33 (24.6)	13 (25.0)		31 (25.8)	17 (21.8)	
None	150 (75.8)	0 (0.0)	10 (83.3)	101 (75.4)	39 (75.0)		89 (74.2)	61 (78.2)	
**Distant metastasis**						0.976			0.912
Present	28 (14.1)	0 (0.0)	2 (22.2)	18 (25.0)	8 (25.8)		16 (24.6)	12 (25.5)	
Absent	84 (42.4)	0 (0.0)	7 (77.8)	54 (75.0)	23 (74.2)		49 (75.4)	35 (74.5)	
**Tumor recurrence**						0.736			0.717
Yes	29 (14.6)	0 (0.0)	3 (33.3)	17 (23.6)	9 (29.0)		16 (24.6)	13 (27.7)	
No	83 (41.9)	0 (0.0)	6 (66.7)	55 (76.4)	22 (71.0)		49 (75.4)	34 (72.3)	

*P value*; Pearson’s χ^2^ test, and Values in bold are statistically significant.

*H-score* histological score.

**Table 5:** The association between membranous DDIT4 expression and clinicopathological features of colorectal cancer (CRC) samples (Intensity of staining and H-score).

Patients and tumor characteristics	Total samples N (%)	Intensity of staining N (%)				*P* value	H-score (cut off = 200) N(%)		*P* value
		0 (Negative)	1+ (Weak)	2+ (Moderate)	3+ (Strong)		Low (≤ 200)	High (> 200)	
**Mean age, years (Range)**	59 (25–88)					0.328			0.143
≤ Mean age	98 (49.5)	0 (0.0)	4 (66.7)	53 (52.5)	41 (46.1)		62 (53.9)	36 (43.4)	
> Mean age	100 (50.5)	2 (100.0)	2 (33.3)	48 (47.5)	48 (53.9)		53 (46.1)	47 (56.6)	
**Gender**						0.781			0.686
Male	104 (52.5)	1 (50.0)	4 (66.7)	50 (49.5)	49 (55.1)		59 (51.3)	45 (54.2)	
Female	94 (47.5)	1 (50.0)	2 (33.3)	51 (50.5)	40 (44.9)		56 (48.7)	38 (45.8)	
(Male/Female)	1.1	1.0	2.0	0.9	1.2		1.0	1.1	
**Mean tumor size (cm)**	5					0.674			0.958
≤ Mean	134 (67.7)	1 (50.0)	3 (50.0)	71 (70.3)	59 (66.3)		78 (67.8)	56 (67.5)	
> Mean	64 (32.3)	1 (50.0)	3 (50.0)	30 (29.7)	30 (33.7)		37 (32.2)	27 (32.5)	
**Tumor differentiation**						0.198			0.817
Well	90 (45.5)	1 (50.0)	4 (66.7)	47 (46.5)	38 (42.7)		54 (47.0)	36 (43.4)	
Moderately	95 (48.0)	0 (0.0)	2 (33.3)	47 (46.5)	46 (51.7)		53 (46.1)	42 (50.6)	
Poorly	13 (6.6)	1 (50.0)	0 (0.0)	7 (6.9)	5 (5.6)		8 (7.0)	5 (6.0)	
**TNM stages**						0.927			0.989
I	31 (15.7)	0 (0.0)	1 (16.7)	17 (16.8)	13(14.6)		18 (15.7)	13 (15.7)	
II	81 (40.9)	0 (0.0)	3 (50.0)	40 (39.6)	38(42.7)		46 (40.0)	35 (42.2)	
III	79 (39.9)	2 (100.0)	2 (33.3)	40 (39.6)	35(39.3)		47 (40.9)	32 (38.6)	
IV	7 (3.5)	0 (0.0)	0 (0.0)	4 (4.0)	3(3.4)		4 (3.5)	3 (3.6)	
**Vascular invasion (VI)**						0.634			0.941
Present	33 (16.7)	0 (0.0)	0 (0.0)	17 (16.8)	16 (18.0)		19 (16.5)	14 (16.9)	
Absent	165 (83.3)	2 (100.0)	6 (100.0)	84 (83.2)	73 (82.0)		96 (83.5)	69 (83.1)	
**Lymph node invasion (LNI)**						0.400			0.724
Involved	84 (42.4)	2 (0.0)	2 (33.3)	43 (42.6)	37 (41.6)		50 (43.5)	34 (41.0)	
None	114 (57.6)	0 (0.0)	4 (66.7)	58 (57.4)	52 (58.4)		65 (56.5)	49 (59.0)	
**Perineural invasion (PI)**						0.647			0.294
Involved	48 (24.2)	1 (50.0)	1 (16.7)	27 (26.7)	19 (21.3)		31 (27.0)	17 (20.5)	
None	150 (75.8)	1 (50.0)	5 (83.3)	74 (73.3)	70 (78.7)		84 (73.0)	66 (79.5)	
**Distant metastasis**						0.878			0.912
Present	28 (14.1)	0 (0.0)	1 (25.0)	14 (25.5)	13 (25.5)		16 (25.4)	12 (24.5)	
Absent	84 (42.4)	2 (100.0)	3 (75.0)	41 (74.5)	38 (74.5)		47 (74.6)	37 (75.5)	
**Tumor recurrence**						0.759			0.568
Yes	29 (14.6)	0 (0.0)	1 (25.0)	13 (23.6)	15 (29.4)		15 (23.8)	14 (28.6)	
No	83 (41.9)	2 (100.0)	3 (75.0)	42 (76.4)	36 (70.6)		48 (76.2)	35 (71.4)	

*P value*; Pearson’s χ^2^ test, and Values in bold are statistically significant.

*H-score* histological score.

**Figure 6:**
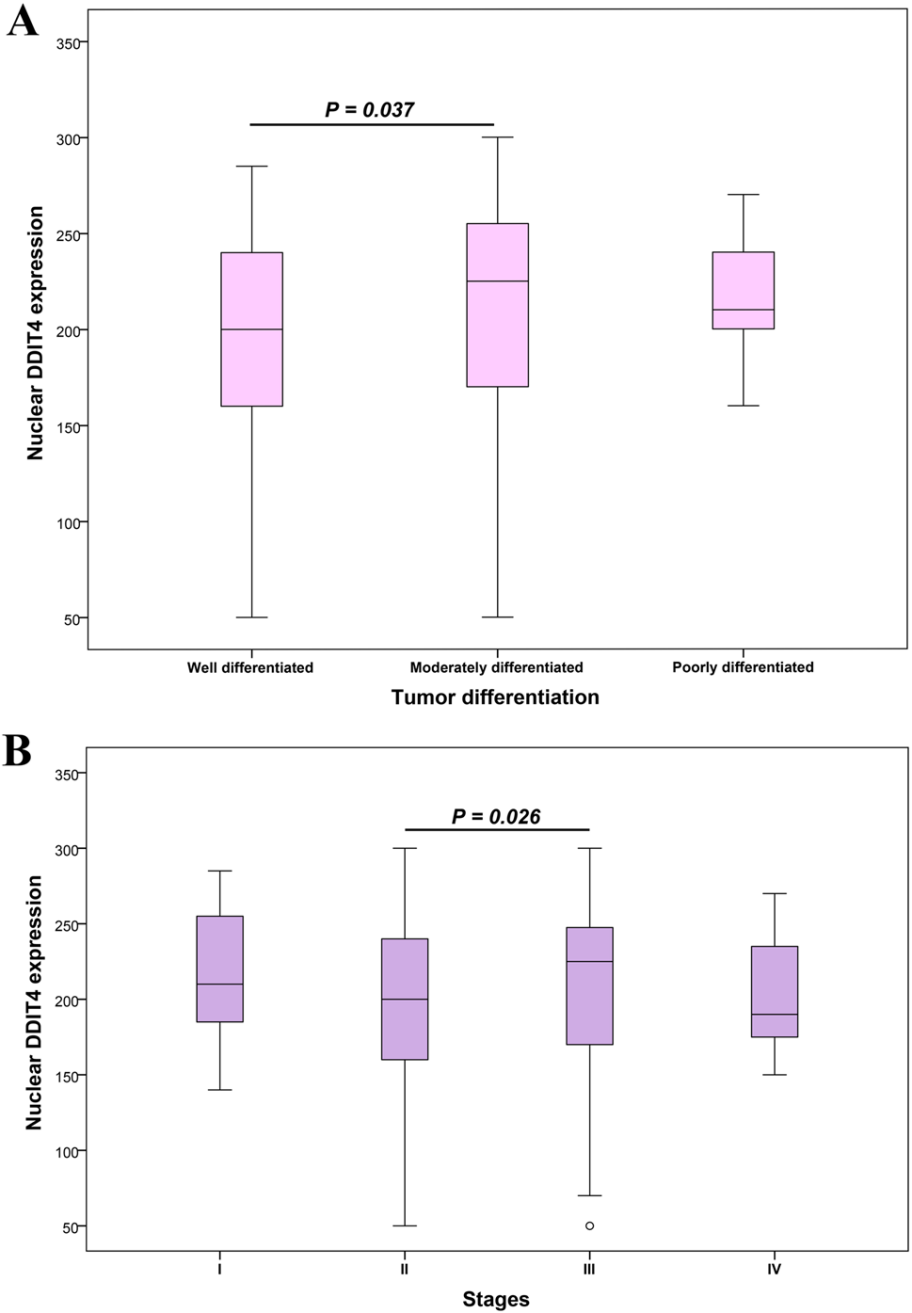
Box plot analysis of DDIT4 expression levels in tumor differentiation groups and TNM stages. The bold line precisely represents median expression levels of DDIT4. (**A**) The result of data analysis showed a statistically significant association in the median nuclear expression of DDIT4 between well and moderately tumor differentiation (*P* = 0.037). (**B**) A statistically significant association was observed in the median nuclear expression of DDIT4 between stages II and III (*P* = 0.026).

The data of the Kruskal–Wallis test exhibited statistically significant differences between the median nuclear expression level of DDIT4 and tumor differentiation groups as well as various TNM stages (I–IV) (*P* < 0.05). Mann–Whitney *U* test also showed a significant difference in median nuclear expression level of DDIT4 between well and moderately differentiated groups of tumor differentiation (*P* = 0.037) (Fig. 6A). Median nuclear expression level of DDIT4 was 225 in moderately differentiated and it was 200 in well differentiated. Moreover, the results of Mann–Whitney *U* test revealed a significant difference in median nuclear expression level of DDIT4 between stage II and stage III (*P* = 0.026). Similarly, median nuclear expression level of DDIT4 was 225 for stage III and 200 in stage II (Fig. 6B). The analysis did not reveal any significant differences in the association between cytoplasmic and membranous expression of DDIT4 with clinicopathological features.

#### Survival outcomes based on expression of DDIT4 in the patients with CRC

**Figure 7:**
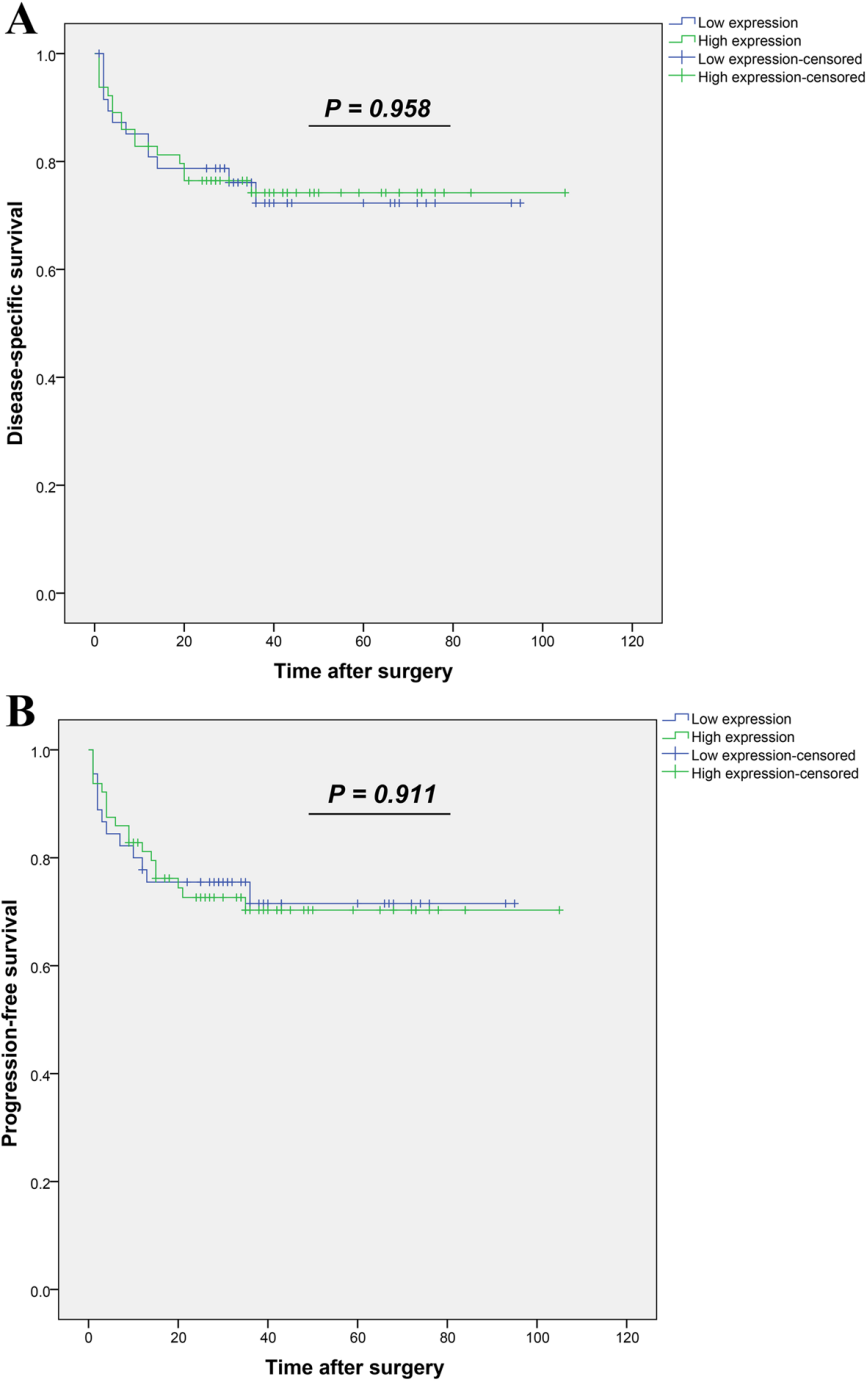
Kaplan–Meier survival analysis according to nuclear expression levels of DDIT4 protein in colorectal cancer (CRC). Log-rank test did not show any significant difference in (**A**) DSS and (**B**) PFS between two groups of CRC patients (high versus low nuclear expression of DDIT4 protein). *DSS*: Disease-specific survival and *PFS*: progression-free survival.

The follow-up data were available for 112 out of 198 patients with CRC in this study. During the follow-up time, disease-related death was reported in 28 patients (14.1%), recurrence and metastasis happened in 29 (14.6%) and 28 (14.1%) patients, respectively, while 79 (39.9%) cases were absent for these two parameters. Median and mean follow-up time for these CRC patients were 34 months (Q1 = 21 and Q3 = 47) (ranging from 1 to 105 months) and 36 (SD = 24), respectively. Survival calculation was applied for disease-specific survival (DSS) and progression-free survival (PFS) terms. Kaplan–Meier survival analysis was implemented to compare survival (DSS and PFS) in two groups of the patients with CRC (high versus low nuclear expression of DDIT4 protein). Results of survival analysis showed no significant association between survival (DSS and PFS) and nuclear expression levels of DDIT4 (Log-rank test; DSS and PFS, P = 0.958, P = 0.911) (Fig. 7A,B).

## Discussion

Regarding study of cancer heterogeneity, biomarkers are valuable that can improve clinical management and the potential for recognition of therapeutic targets in drug development for CRC^39^. In this regard, development of markers and multiple therapeutics has led to an increase in survival up to 3 years for the patients with CRC at advanced stages of disease that is still not satisfactory^40^. For recognizing biomarkers in CRC, three series of the GEO were analyzed and common upregulated DEGs of these series were screened. Results of the PPI network and enrichment analyses determined hub genes that a few of them including DDIT4 were involved in ‘microRNAs in cancer’, a pathway in CRC disease based on the KEGG DISEASE Database. Moreover, KEGG pathway enrichment analysis revealed that DDIT4 participates in PI3K-Akt/mTOR signaling pathways as important regulators for properties of pluripotent stem cells^41^. These signaling pathways are responsible for various functions and biological processes in cell, such as proliferation, differentiation, and migration^42^. It is noteworthy that the defect of PI3K-Akt/mTOR signaling pathways has been reported in development and advances of CRC^43^. Our findings reviewing literature indicated protein expression of DDIT4 in CRC clinical samples has received much less attention. Also, a higher expression of DDIT4 in mRNA level was observed in fresh CRC tumors compared to adjacent normal tissue samples in our previous study^35^. To the best of our knowledge, this study is the first to investigate of protein levels and clinical significance of DDIT4 in the patients with CRC by TMA-based IHC method.

DDIT4 is an attractive potential target for therapeutic approach in cancer^12^ silencing of which leads to sensitization of tumor cells to cancer treatment and drugs in in-vitro and in-vivo studies^19,22,30,33^. Inconsistent roles have been reported for DDIT4 in cell death and carcinogenesis so that, upregulated expression of DDIT4 occurs due to DNA-damaging agents via nuclear p53 manner that inhibits mTORC1^44,45^ and as a result can increase cell death^21,44^. On the other hand, high expression of DDIT4 can protect human cancer cells from hypoxia-induced cell death^19,46,47^ through stabilizing HIF1α in downstream of the suppressed mTOR pathway which followed by occurs increased cell survival and tumor growth^47^. Moreover, studies have indicated a relationship between expression of DDIT4 and an increase in expression of BCL2 as anti-apoptotic protein and also changes in p53 phosphorylation reducing apoptosis^12,22^.

In-silico findings have shown high expression of DDIT4 in several cancers that is significantly associated with a worse prognosis^48^. Our results using three GEO series illustrated the upregulated expression of DDIT4 in CRC tissues compared to adjacent normal tissues, corroborating with the previous study that indicated overexpression of DDIT4 as prognosis biomarker in the patients with CRC using bioinformatics tools^49^. For further considering this marker, expression level of DDIT4 protein was investigated in 198 tissue samples from patients after surgery. Expression levels of DDIT4 were noticed in nucleus, cytoplasm, and plasma membrane substantiating the evidence in the literature^31,50,51^, and COMPARTMENTS database. Previous results have demonstrated localization of DDIT4 in nucleus and cytoplasm of the cells before activation while its translocation to plasma membrane was observed during activation and probably, it becomes disabled by this mechanism^51^.

The evaluation of DDIT4 staining was obtained for each subcellular location in cells in a series of CRC tissues with a range of intensities from weak to strong. Our analysis showed a statistically higher nuclear expression of DDIT4 compared to adjacent normal tissues in CRC, which is in line with the study by Chang et al. that observed high nuclear expression of DDIT4 in OC tissues compared to normal tissues^31^. This result also validates the finding of a previous experiment that identified higher levels of DDIT4 protein in 10 CRC tissues compared to adjacent normal tissues by the western blot method^47^. In addition, results of the population study confirmed and supported upregulation of DDIT4 expression in our bioinformatics analysis. We observed positive correlates of nuclear DDIT4 expression with the TNM stages and tumor differentiation. Notably, TNM staging and tumor differentiation are generally considered as traditional and important prognostic factors advance in which is associated with worse outcome for the patients with CRC^4,52–54^. Generally, in solid tumors, an immature tumor is more aggressive than a tumor with a differentiated cell phenotype^55^. Interestingly, median nuclear expression of DDIT4 was significantly higher in more advanced stage (stage III) compared to stage II, showing the association of nuclear expression of DDIT4 protein with aggressiveness of CRC. In addition, a significant association was found between the increased expression of DDIT4 and the decreased tumor differentiation so that, the patients with CRC who had moderately differentiated tumor cells showed higher nuclear expression of DDIT4 rather than cases with well differentiated tumor cells. This result is in line with the study by Chen et al. that showed overexpression of DDIT4 may maintain seminomas in an undifferentiated state^56^. Furthermore, there was no significant association between cytoplasmic and membranous expression of DDIT4 with clinicopathological features. Therefore, our results suggested that nuclear expression of DDIT4, rather than its cytoplasmic or membranous expression is related to advances of malignancy and progression in CRC. There was no significant association between expression of DDIT4 and survival in the patients with CRC, which is consistent with earlier findings on GC^22^ but is contrary to the previous published studies on OC^31,32^. Given that our findings are based on follow-up time, it can be mentioned that a long-term follow-up may plausibly alter prognostic value of DDIT4 expression.

In CRC samples, more strong intensity and higher median expression of DDIT4 in nuclear staining for tumor tissues might be due to the role of DDIT4 in the cancer cells҆ nucleus. It has been proposed that DDIT4 may have different functions in subcellular localization^31,50^; nevertheless, little information exists about nuclear expression of DDIT4. Despite several questions regarding DDIT4 functions in different cell locations, DDIT4 is a key player within the mTOR pathway that is critical for the cells. It is important to note that inhibition of mTOR pathway leads to the increased expression of cancer stem cell (CSC) markers and CSC populations^57^, CSCs are a rare subpopulation of cancer cells within tumors with stemness, self-renewal, quiescence, and tumorigenicity properties that are resistant to cancer therapy^58,59^. Studies have indicated that expression of DDIT4 promotes stemness markers and is related to features of CSC^60–62^. Drug resistance and therapeutic failure of tumor cells seems to be closely related to various properties and indispensable functions of CSCs, such as quiescence, overexpression of anti-apoptotic proteins, and the ability of DNA repair^63,64^.

In conclusion, findings of this study highlighted overexpression of DDIT4 as a biomarker in CRC tissues especially in nucleus of tumor cells. Overexpression of DDIT4 protein may indicate more aggressive tumor behavior and more advanced disease in the patients with CRC. Although, nuclear expression of this marker has been observed in other tumors, function of the nuclear expression of DDIT4 protein has not been reported in the literature. Accordingly, evidence indicates that DDIT4 is a potential therapeutic marker. Therefore, there is a need to increase the knowledge regarding the role of DDIT4 in cell proliferation pathways and progression of cancer that may lead to improvement of prognosis and developing agents for targeted treatment.

## Methods

### Bioinformatics analysis

#### Microarray data source and screening for differentially expressed genes (DEGs)

##### Data source

Primary search was performed on the Gene Expression Omnibus (GEO) database^65^ of the National Center for Biotechnology Information (NCBI) using keywords of "Colorectal Neoplasms" OR "Colorectal Cancer" OR "Colorectal Tumor" as search terms. Obtained GEO series of primary search were limited to expression profiling by array, homo sapiens, tissues, and publication dates [1 year (OCT 2018–2019)]. The samples and experiments҆ condition of series were closely checked in order to minimize heterogeneity for selection of expression profiles. Also, the last series of colon cancer without time limitation was added because colon cancer is more common than rectal cancer^1^. Finally, three gene expression series including –GSE74602, GSE110223, and GSE110224—were selected from the GEO. Among series, GSE74602 had used 30 tumors and 30 adjacent normal colon samples based on the Agilent GPL6104 platform (Illumina humanRef-8 v2.0 expression beadchip). GSE110223 was based on GPL96 platform ([HG-U133A] Affymetrix Human Genome U133A Array) that included 13 CRC tumors and 13 adjacent normal samples. While GSE110224 consisted of 17 CRC tumors and 17 adjacent normal samples with GPL570 platform ([HG-U133_Plus_2] Affymetrix Human Genome U133 Plus 2.0 Array).

##### Identification of DEGs

The classification of CRC samples for our analysis was only based on their tumor and adjacent normal tissues, because our CRC patients in the population study section was lacking molecular subtypes of CRC. Expression of the genes was compared between tumors and adjacent normal tissues using the GEO2R online analysis tool (http://www.ncbi.nlm.nih.gov/geo/geo2r) for each series. The DEGs in CRC tissues base cut-off criteria (logFC ≥  ± 1.0 and adjusted *P* value < 0.05) of each series were obtained and visualized by volcano plot using OriginPro 2019 (OriginLab Corporation, Northampton, MA, USA). Common upregulated DEGs in CRC tissues were extracted from three series for subsequent analysis by Venn diagram analysis in OriginPro 2019 software. In order to recheck the common upregulated genes obtained in this method, the results were compared with common DEGs obtained by MINT method. MINT is a powerful approach to solve the integrative classification framework by combining multiple independent studies and is a part of the mixOmics R CRAN package^66^. Therefore, 3 series of GEO were merged and detected common DEGs for them by MINT method in R platform.

#### Generation of PPI network and extraction of hub genes

##### PPI network

For finding more data about common upregulated DEGs and their interactions with other proteins in cellular activities, PPI network construction was performed using the International Molecular Exchange Consortium (IMEx) in Cytoscape software^67^. IMEx includes some databases, such as IntAct, DIP, HPIDB, MINT, and UniProtKB/Swiss-Prot without redundant set of interactions checking data quality and ensuring consistency between all the series^68^.

##### Hub genes

The genes with high connectivity degree (> 75 percentile for genes with degree > 10) were selected from PPI network in Cytoscape software by cyto-Hubba plug-in^69^. Among the obtained genes with high connectivity degree for this network, only the genes belonging to our common upregulated DEGs were selected as hub genes by Venn diagram.

#### Pathway and functional enrichment analysis for hub genes

The KEGG pathway analysis for all the hub genes was done in order to scrutinize the biological pathways in which these genes are involved. Then, gene ontology (GO) term enrichment analysis was performed in domains of molecular function (MF) and biological process (BP) only for the hub genes involved in pathways with *P* value < 0.05. Moreover, pathways similar to those mentioned in CRC disease according to the KEGG DISEASE Database were considered in order to select a biomarker^70^. These analyses were performed in Cytoscape software using ClueGO plug-in assisting the GO/pathway analysis and visualizing functionally grouped terms in networks and graphs^71^. Finally, one hub gene was selected for evaluation of protein expression levels using the IHC method in CRC tissue samples. Subcellular localization of this maker was investigated in COMPARTMENTS database^72^.

### Population study

#### Collection of tissue samples and TMA construction

##### Patients

FFPE blocks as well as hematoxylin and eosin (H&E)-stained slides of 198 CRC and 39 adjacent normal tissues from patients that had undergone tumor resection were included in this study. These samples were collected from three university hospitals-Hashemi Nejad, Firoozgar, and Hazrate Rasoole Akram in Tehran, Iran during 2012–2018. Patients had not received any relevant radiotherapy, chemotherapy, or immunotherapy before surgery. Clinicopathological features including age, gender, tumor size, tumor differentiation, TNM stage, vascular invasion (VI), lymph node invasion (LNI), perineural invasion (PI), distant metastasis, and tumor recurrence were obtained by reviewing clinicopathological records for each sample defined according to the American Joint Committee on Cancer (AJCC) in 2018^52^.

Outcome information of the patients after surgery was also recorded. Disease-specific survival (DSS) was described as the time from initial surgery to the date of CRC-related death. Progression-free survival (PFS) was determined as the time between the primary surgery and the last follow-up interview if the patient with CRC displayed no evidence of disease, recurrence, or metastasis.

##### TMA construction

TMA blocks were provided as described previously^73^. In brief, the most representative areas of the tumor and normal cells in different parts of tissue samples were selected by checking H&E slides. Then, a core of 0.6 mm diameter was separated from the selected areas in each donor block and was transferred to a new recipient paraffin block using a precision arraying instrument (Tissue Arrayer Minicore; ALPHELYS, Plaisir, France). In the current study, 3 cores of the selected areas randomly from each tissue sample were transferred and scored individually. These TMA blocks were built of different cores for each sample. TMA slides were obtained by cutting sections of TMA blocks, to a thickness of about 4 μm, which were transferred to an adhesive‑coated slide system (SuperFrost Plus, Thermo Scientific™, Germany). Mean expression of 3 cores was calculated from each tissue sample in order to increase accuracy and validity of the data analysis. The TMA-based IHC method is robust with respect to sampling that could increase this accuracy similar to conventional tissue sections, even analysis of 2 cores increased accuracy by more than 95%^74,75^.

#### Applying IHC method on TMA slides and immunostaining system scoring

##### IHC procedure

Immunostaining of TMA sections for DDIT4 was performed as described previously^76^. The slides were deparaffinized after 30 min at 60 °C and were rehydrated in xylene and serial ethanol dilutions, respectively. H_2_O_2_ (3%) was used for blockage of endogenous peroxidase on tissue sections for 20 min at room temperature. Following three times washing the tissue sections, antigen retrieval was carried out by covering the tissues in citrate buffer (pH 6.0) for 10 min in an autoclave. Subsequently, tissue sections with primary antibody specific for DDIT4 (1:80 dilution, Biorbyt, Cambridge, UK) were incubated overnight at 4 °C. Furthermore, rabbit immunoglobulin IgG (Invitrogen, Thermo Fisher Scientific, Waltham, MA, USA) was applied at a dilution of 1:500 for isotype control test. For visualizing bound antibody-antigen, TMA slides were incubated with the secondary antibody (EnVision Kit, Dako, Glostrup, Denmark) for 30 min and then 3, 30-diaminobenzidine as chromogen (DAB, Dako, Glostrup, Denmark). The hematoxylin (Dako, Glostrup, Denmark) was used a counterstain for all the slides. Finally, the tissues slides were dehydrated in alcohol, and were cleared in xylenes until scoring by pathologists. The normal liver tissue was applied as positive control while Tris-buffered saline (TBS) was replaced by the primary antibody as the negative control per each run of the test.

##### Evaluation of immunostaining

Positive cells for staining of DDIT4 were assessed using a semi-quantitative scoring at 40X magnifications by two pathologists (Z.HS. and M.M.) who were blinded to pathological parameters and the patients҆ outcomes. DDIT4 staining intensity was scored in a 4-point scale (staining intensity scores: absent: 0, weak: 1, moderate: 2, or strong: 3) and percentage was estimated according to < 25, 25–50%, 51–75%, and > 75% of positive tumor cells. In calculation of the scores, H-score was obtained for each core through multiplying the intensity score by percentage of the stained cells ranging from 0 to 300^77^. The final H-scores were calculated from average of three score spots for each sample and were classified into two groups (low or high expression) according to median expression of DDIT4.

#### Statistical analysis

The Chi-square and Spearman’s correlation tests were used to analyze the association and correlation between expression of DDIT4 and clinicopathological features. The Kruskal–Wallis and Mann–Whitney *U* tests were applied for comparison of DDIT4 expression between groups. DSS and PFS were calculated using the Kaplan–Meier method by the log-rank test to compare survival outcomes between patient groups (low and high expression of marker). The data were expressed as mean with standard deviation (SD) and median with quartile (Q1, Q3). All the statistical analyses for the obtained clinical data were performed using SPSS v.22.0 software (IBM Corp., Armonk, NY, USA) and P-value less than 0.05 was considered as statistically significant.

### Ethical approval

This study was approved by the Iran University of Medical Sciences Research Ethics Committee (IR.IUMS.REC.1398.1043). All procedures performed in this study were in accordance with the 1964 Helsinki Declaration and its later amendments. Also, informed consent was obtained from all individual participants included in the study at the time of sample collection in accordance with the above-mentioned ethical standards.

## Supplementary Information

Supplementary Figures. MINT sPLS-DA plots for differentially expressed genes (DEGs) in CRC by MINT method as well aspathways related to colorectal cancer (CRC) recorded on the KEGG DISEASE Database.

Supplementary Table 1. Lists of up and down-regulated genes from GSE74602, GSE110223 and GSE110224, common upregulatedDEGs, high connectivity degree genes and hub genes as well as the obtained DEGs from merged 3 series by MINTmethod.

Supplementary Table 2. Results of enrichment analysis by ClueGO

## Data Availability

The analyzed data during the current study are available from the corresponding authors on reasonable request.
