# Supplementary material for: High expression of DNA damage-inducible transcript 4 (DDIT4) is associated with advanced pathological features in the patients with colorectal cancer
Source: Sci Rep. 2021 Jul 1;11:13626. doi: 10.1038/s41598-021-92720-z (PMC8249407; doi:10.1038/s41598-021-92720-z)
Supplement: Supplementary file 1 — MINT sPLS-DA plots for differentially expressed genes (DEGs) in CRC by MINT method as well aspathways related to colorectal cancer (CRC) recorded on the KEGG DISEASE Database. [file 41598_2021_92720_MOESM1_ESM.pdf]

High Expression of DNA Damage-Inducible Transcript 4 (DDIT4) Is Associated with Advanced Pathological Features in the Patients with Colorectal Cancer

Fahimeh Fattahi, Leili Saeednejad Zanjani, Zohreh Habibi Shams, Jafar Kiani, Mitra Mehrazma, Mohammad Najafi, Zahra Madjd

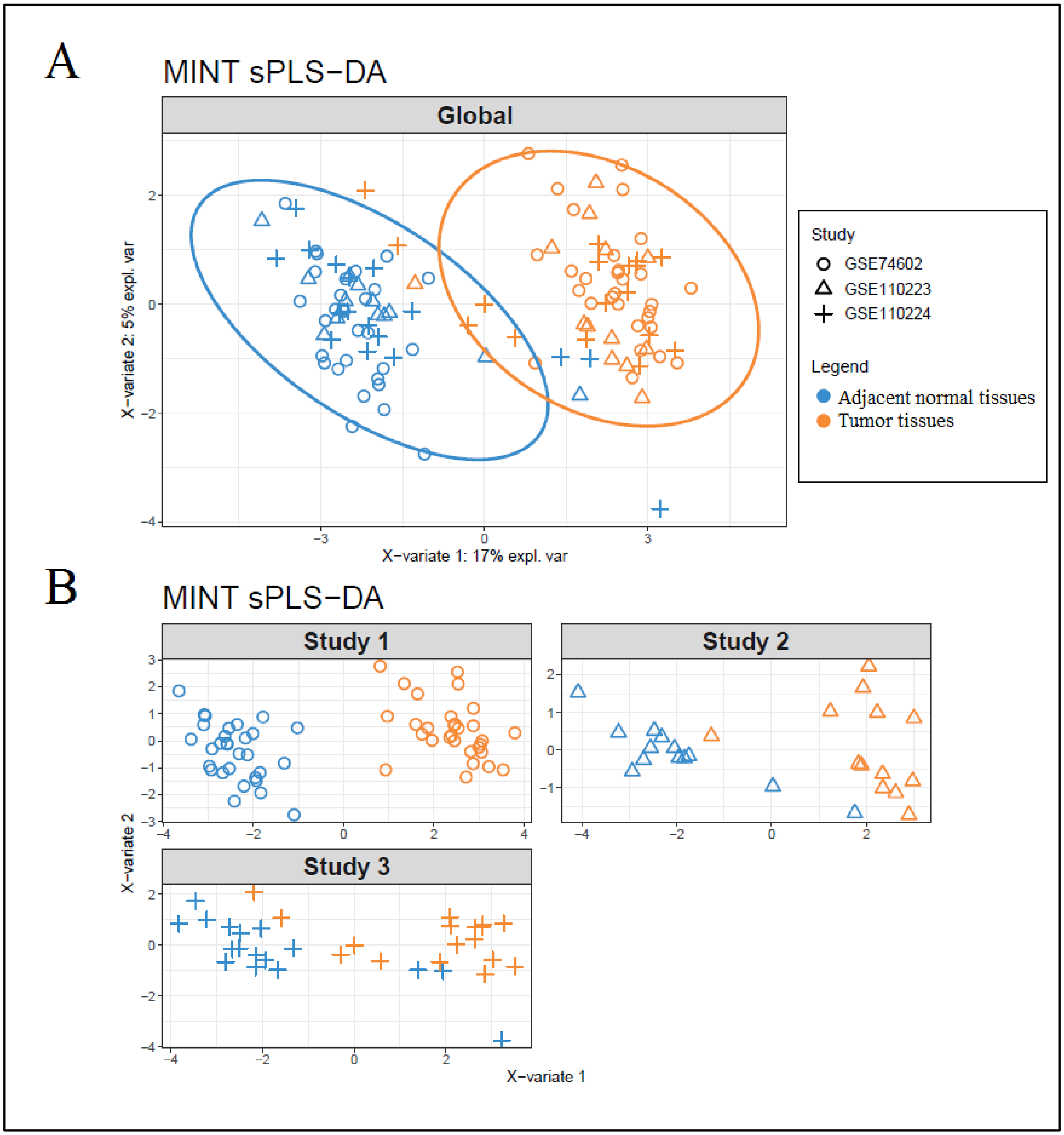

Supplementary Fig.S1. MINT sPLS-DA plots for differentially expressed genes (DEGs) in CRC by MINT method. MINT sPLS-DA plots for **A)** DEGs in tumor tissues of merged three study (GSE74602, GSE110223, and GSE110224) from GEO and **B)** DEGs in tumor tissues of GSE74602, GSE110223, and GSE110224 separately were shown by MINT method in R platform.

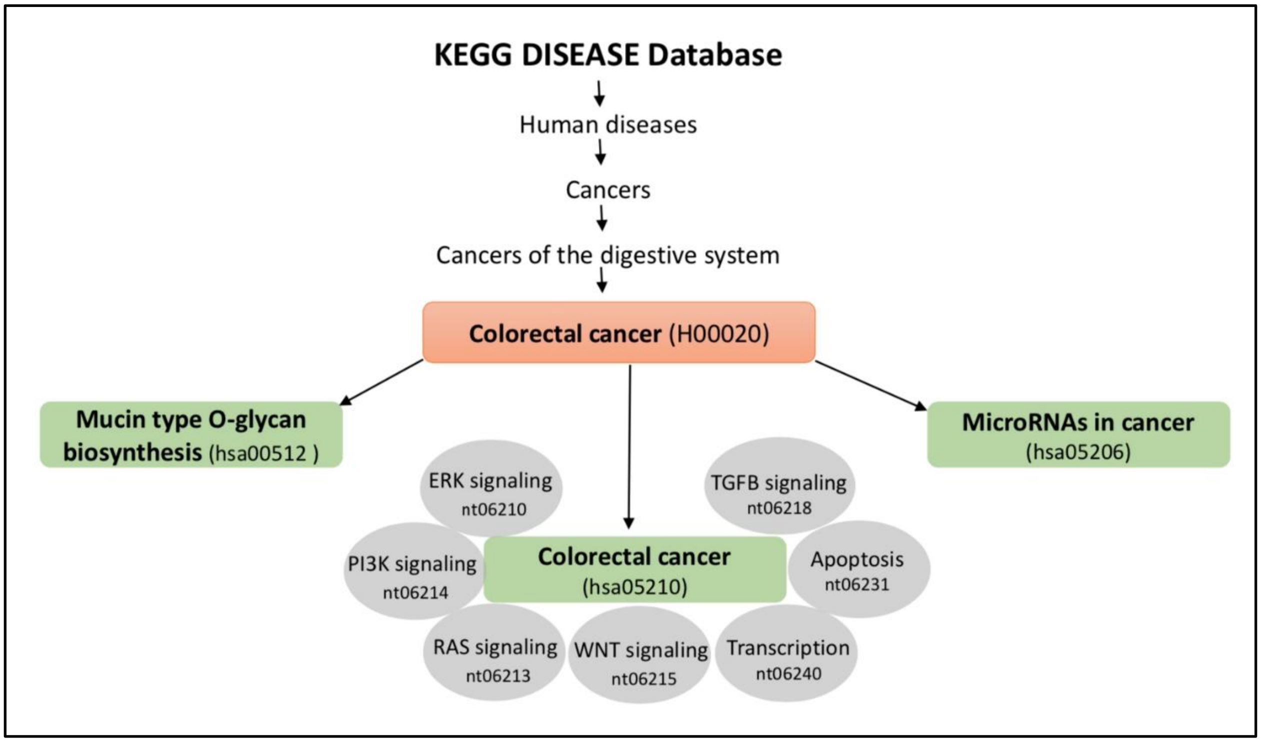

Supplementary Fig.S2. Pathways related to colorectal cancer (CRC) recorded on the KEGG DISEASE Database. In this flowchart, the access path to CRC pathways on the KEGG DISEASE Database is summarized.
